# Decoding Fibroblast Diversity Associated with the Postnatal Loss of Cardiac Regenerative Capacity

**DOI:** 10.3390/ijms27062709

**Published:** 2026-03-16

**Authors:** Parisa Aghagolzadeh, Vincent Rapp, Mohamed Nemir, Felix Mahfoud, Marijke Brink, Thierry Pedrazzini

**Affiliations:** 1 Experimental Cardiology Unit, Division of Cardiology, Department of Cardiovascular Medicine, University of Lausanne Medical School, 1011 Lausanne, Switzerland; 2 Cardiobiology, Department of Biomedicine, University of Basel and University Hospital Basel, 4031 Basel, Switzerland; 3 Translational Cardiology, Department of Biomedicine, University of Basel and University Hospital Basel, 4031 Basel, Switzerland; 4 Cardiovascular Research Institute Basel (CRIB), University Heart Center, University Hospital Basel, 4031 Basel, Switzerland; 5 Clinic for Cardiology, University Heart Center, University Hospital Basel, 4031 Basel, Switzerland; 6 School of Cardiovascular and Metabolic Medicine and Sciences, and MRC/BHF Centre of Research Excellence in Advanced Cardiac Therapies, King’s College London, London WC2R 2LS, UK

**Keywords:** cardiac fibroblasts, postnatal cardiac maturation, single-cell RNA sequencing (scRNA-seq), myocardial infarction (MI)

## Abstract

The mammalian heart rapidly loses regenerative capacity after birth and responds to myocardial infarction (MI) with scar formation and development of interstitial fibrosis. Cardiac fibroblasts orchestrate extracellular matrix (ECM) remodeling and cell–cell communication during development and injury; however, how fibroblast heterogeneity and fibroblast communication networks differ between regenerative neonatal and non-regenerative adult hearts remains incompletely defined. We performed scRNA-seq analysis on metabolically active CD45^−^/CD31^−^ nonmyocyte cells from the left ventricles of normal neonatal (P3) and adult (P84) mice to probe heterogeneity in a cardiac fibroblast-enriched population. We identified five transcriptionally distinct cardiac fibroblast subclusters (CF0-CF4) demonstrating different distributions across ages, including an adult-enriched immune/complement-associated program (CF0); an ECM structural-associated program present across ages (CF1); and neonatal-enriched contractile/ECM-remodeling (CF2), Wnt-modulating matrix-regulatory (CF3), and proliferative (CF4) programs. Matrisome category scoring revealed age-dependent divergence in ECM programs: neonatal fibroblasts showed higher enrichment of core matrisome components (particularly collagens and proteoglycans), whereas adult fibroblasts were relatively enriched for matrisome-associated categories, including ECM regulators and secreted factors. Ligand–receptor inference using CellChat demonstrated a broad reduction in fibroblast–fibroblast interaction strength and information flow in adult networks, and adult-enriched signaling was dominated by immune/chemotactic pathways. Finally, projection of subcluster marker programs onto an independent bulk RNA-seq dataset of cardiac fibroblasts 3 days after MI revealed that adult injury partially recapitulates neonatal-associated programs, including activation of the contractile/ECM-remodeling program (CF2) and robust induction of a cell-cycle-associated program (CF4), but lacks an additional neonatal-specific injury program associated with the Wnt-modulating subset (CF3), which was weakly induced or absent in adults. This cardiac fibroblast-enriched single-cell study defines age-dependent fibroblast states, ECM specialization, and communication network architecture that distinguish regenerative neonatal from non-regenerative adult hearts. It also provides a framework to interpret divergent stromal responses after MI and to prioritize fibroblast programs for regenerative and anti-fibrotic strategies.

## 1. Introduction

The mammalian heart undergoes a developmental transition from a temporarily regenerative state in early life to a largely non-regenerative state in adulthood. Whereas neonatal mammalian hearts can restore structure and function after injury, adult hearts respond to myocardial infarction (MI) with permanent scar formation, adverse remodeling, and progressive functional decline [1,2]. Despite major advances, our understanding of the cellular programs accompanying the postnatal loss of cardiac regenerative capacity remains incomplete, and it is unclear how the stromal compartment—particularly cardiac fibroblasts—shapes regenerative versus fibrotic outcomes.

Cardiac fibroblasts are central organizers of myocardial homeostasis and repair [3,4]. Beyond their canonical role as the dominant source of ECM [5], fibroblasts integrate mechanical and inflammatory signals, coordinate cross-talk with cardiomyocytes and vascular cells, and dynamically adopt diverse transcriptional states during development and injury [6,7,8,9]. Single-cell studies have established that fibroblast populations are heterogeneous in the adult heart and undergo marked activation after MI in adults [10]. However, how fibroblasts are configured in neonatal hearts to create a permissive environment for regeneration remains unexplored. Moreover, how nonmyocyte cell communication networks evolve from neonatal to adult stages is not well understood. Under these conditions, the balance between adaptive and maladaptive remodeling depends not only on fibroblast abundance but also on the identity of fibroblast subpopulations and their roles as signal generators and integrators of stress signals.

Here, we conducted a cardiac fibroblast-enriched single-cell transcriptomic analysis to evaluate nonmyocyte cell populations in regenerative neonatal (P3) and non-regenerative adult (P84) hearts. We identified distinct subclusters of cardiac fibroblasts within CD45^−^/CD31^−^ nonmyocytes that were differentially enriched in the neonatal vs. the adult heart. To systematically characterize ECM-related specialization, we quantified matrisome category enrichment [11] across fibroblast subclusters and across age, revealing opposing ECM program architectures between neonatal versus adult fibroblast landscapes. Because stromal function is also shaped by intercellular signaling, we then mapped fibroblast subcluster communication networks using ligand–receptor inference and compared predicted signaling architecture between neonatal and adult hearts [12]. Finally, to connect healthy fibroblast reference states to injury responses, we projected subcluster marker programs onto an independent bulk RNA-seq dataset of neonatal and adult cardiac fibroblasts 3 days after MI [1]. This approach identified both shared and age-specific components of the fibroblast injury response.

## 2. Results

### 2.1. Single-Cell Transcriptomic Profiling of Cardiac Fibroblasts Across Neonatal and Adult Hearts

**Figure 1:**
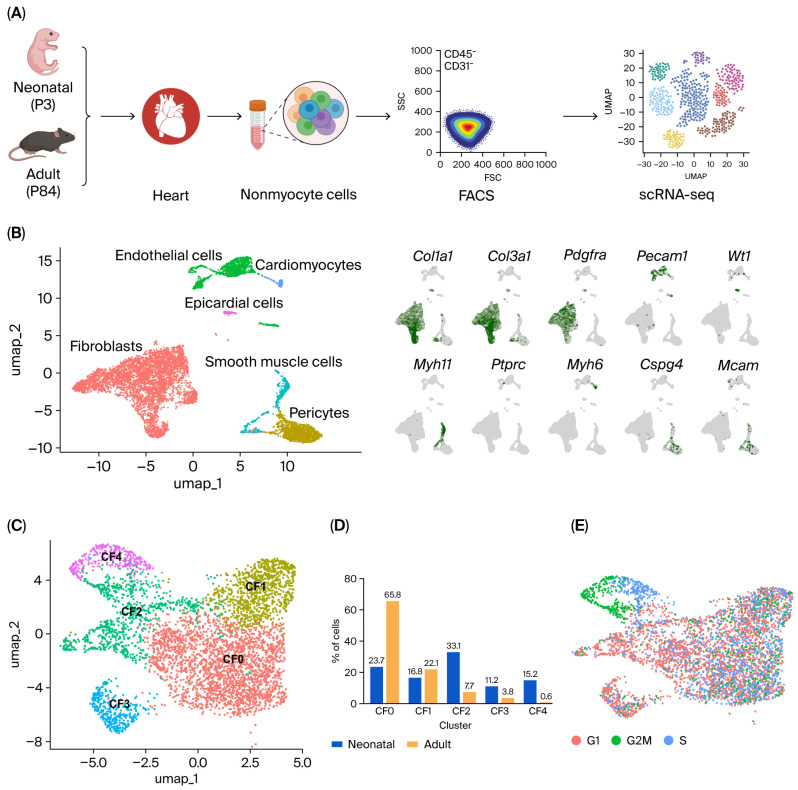
Single-cell transcriptomic profiling and reclustering of cardiac fibroblasts in healthy neonatal and adult hearts. (**A**) Experimental workflow. Left ventricular tissue from neonatal (P3) and adult (P84) mice was enzymatically dissociated. Metabolically active, viable nonmyocytes were isolated by FACS and gated as CD45^−^/CD31^−^ to minimize leukocyte and endothelial contamination prior to droplet-based scRNA-seq. No positive-selection marker was used for fibroblasts to preserve heterogeneity. (**B**) UMAP of the integrated dataset colored by annotated cell populations. Feature plots show expression of canonical marker genes used for annotation: fibroblasts (*Col1a1*, *Col3a1*, *Pdgfra*), endothelial cells (*Pecam1*), epicardial cells (*Wt1*), smooth muscle cells (*Myh11*), cardiomyocytes (*Myh6*), pericytes (*Cspg4*, *Mcam*), and leukocytes (*Ptprc*). (**C**) UMAP of reclustered cardiac fibroblasts showing five fibroblast subclusters (CF0–CF4) and corresponding hierarchical relationships between subclusters (dendrogram). (**D**) Proportion of neonatal and adult fibroblasts within each subcluster (each group sums to 100%). (**E**) UMAP showing the cell-cycle phases based on the expression of the cell-cycle gene collection.

To compare cardiac fibroblast transcriptomes between neonatal and adult hearts and to assess age-dependent fibroblast heterogeneity, we performed scRNA-seq on left ventricular cells from healthy mice at P3 (neonatal) and P84 (adult). To increase the representation of CFs in the cell suspension, we enriched the starting population prior to single-cell sequencing. After enzymatic dissociation, cardiomyocytes were largely depleted through sedimentation and centrifugation (adult cells) or exclusion during sorting (neonatal cells). CD45^−^/CD31^−^ viable cells (Calcein^+^/DAPI^−^) were then isolated via fluorescence-activated cell sorting (FACS) to remove leukocytes and most endothelial cells (Figure 1A; Supplementary Figure S1). No positive selection marker was used for CFs to preserve fibroblast heterogeneity and avoid bias toward predefined subsets.

Following quality control, 3345 P3 cells and 4697 P84 cells were included in the analysis. The expression matrix contained 35,866 genes (RNA assay features). Data integration, unsupervised clustering, and UMAP identified a dominant fibroblast population expressing *Pdgfra*, a characteristic marker of quiescent fibroblasts, and ECM genes such as *Col1a1* and *Col3a1*. Additionally, smaller clusters corresponding to pericytes (*Cspg4*, *Mcam*), smooth muscle cells (*Myh11*), and epicardial cells (*Wt1*) were detected (Figure 1B). A small cluster of contaminating CD31^+^ (*Pecam1^+^*) endothelial cells was also detected, whereas CD45+ (*Ptprc*^+^) cells were not observed, consistent with effective leukocyte depletion following FACS. To define fibroblast heterogeneity in regenerative vs. non-regenerative hearts, we bioinformatically isolated the fibroblast compartment in our scRNA-seq dataset and performed high-resolution reclustering. This identified five cardiac fibroblast subclusters (CF0–CF4) (Figure 1C). Throughout the manuscript, we use “cardiac fibroblasts” to refer to the overall fibroblast population and “CF0–CF4” to refer specifically to the defined fibroblast subclusters. After excluding non-fibroblast clusters and fibroblast-specific reclustering, 4784 fibroblasts were retained for downstream analysis. To further validate cell identity after fibroblast-specific reclustering, we examined canonical fibroblast and non-fibroblast markers in the pooled reclustered fibroblast dataset (Supplementary Figure S2). Residual expression of non-fibroblast markers was minimal, whereas canonical fibroblast markers remained broadly expressed. The subclusters formed distinct transcriptional states on the UMAP embedding. Fibroblast composition differed between groups (Figure 1D). CF0 was strongly enriched in adult hearts (65.8% in adult vs. 23.7% in neonatal). In contrast, CF2 (33.1% in neonatal vs. 7.7% in adult), CF3 (11.2% vs. 3.8%), and CF4 (15.2% vs. 0.6%) were enriched in neonatal hearts, whereas CF1 represented an intermediate fraction in both groups (16.8% neonatal vs. 22.1% adult) (Figure 1D). Cell-cycle projection further indicated enrichment of cycling (S/G2M) cells within CF4, whereas other CF subclusters were predominantly in G1 (Figure 1E).

**Figure 2:**
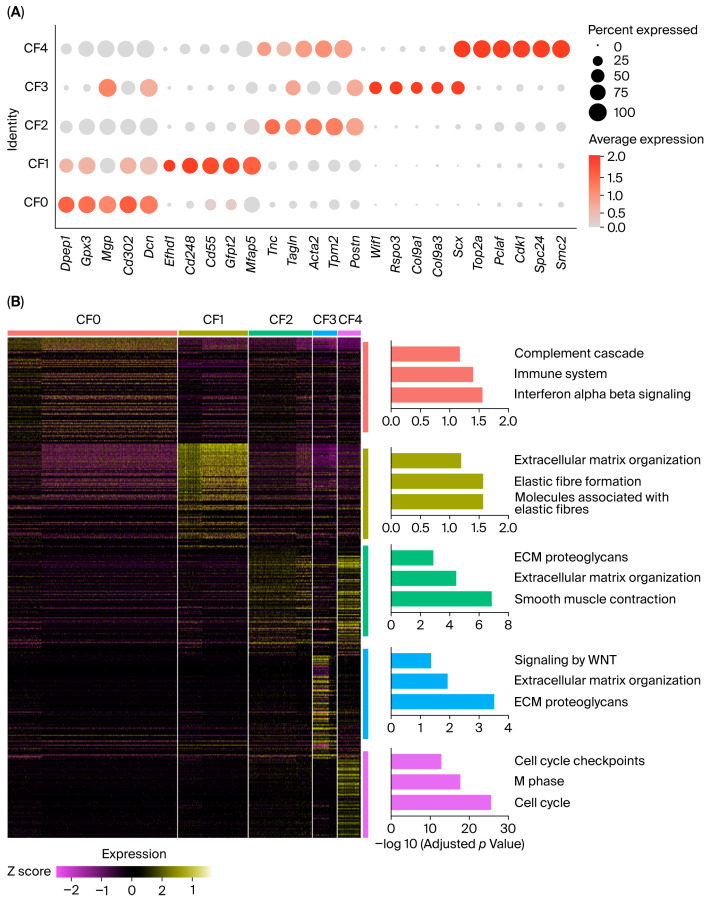
Marker genes and pathway enrichment define functional programs across cardiac fibroblast subclusters. (**A**) Dot plot showing the top 5 marker genes for each CF subcluster (CF0–CF4) (dot size indicates percent of cells expressing each gene; color indicates average expression). Extended marker lists (top 50 genes per subcluster) are provided in Supplementary Table S1. (**B**) Heatmap of the top 50 marker genes per CF subcluster, with Reactome pathway enrichment analysis performed using Enrichr on the top 50 markers for each subcluster. Bar plots show the top enriched Reactome terms per subcluster; bars indicate −log10 (adjusted *p* value), with *p* value adjustment performed using the Benjamini–Hochberg method.

Marker gene analysis supported distinct transcriptional programs across CF subclusters (Figure 2A shows the top 5 marker genes per subcluster; Supplementary Table S1 lists the top 50 markers per subcluster). CF0 showed high expression of genes, including *Dcn*, *Gpx3*, *Mgp*, and *Dpep1*, indicating a homeostatic, ECM maintenance fibroblast program. CF1 was marked by enrichment of *Cd248*, *Cd55*, *Gfpt2***,** and *Mfap5*, indicating an ECM-associated gene program. CF2 was characterized by a contractile and ECM-remodeling program with *Postn***,** *Acta2***,** *Tagln***,** and *Tnc*. CF3 was distinguished by *Wif1* and *Rspo3*, together with *Scx* and collagen IX genes (*Col9a1***/***Col9a3*), defining a Wnt-modulating fibroblast subset. In particular, CF3 showed high expression of *Wif1*, encoding a secreted canonical WNT pathway inhibitor implicated in cardiac repair after myocardial infarction [13,14], and *Fmod*, which inhibits fibrillogenesis and sequesters pro-fibrotic TGF-β within the ECM [15]. Together, these features support a fibroblast state associated with potential anti-fibrotic properties. CF4 displayed a proliferative signature defined by cell-cycle and mitotic genes (*Top2a*, *Cdk1*, *Aurkb*, *Pclaf*, and *Mki67*), which was consistent with the cell-cycle projection showing relative enrichment of cycling (S/G2M) cells in this cluster, whereas other CF subclusters were predominantly in G1 (Figure 1E).

To functionally annotate these subcluster programs, we performed pathway enrichment analysis (Reactome) using Enrichr on the top 50 marker genes for each CF subcluster (Figure 2B; bars indicate −log10 adjusted *p* value). CF0 markers were enriched in immune response-associated pathways, including complement cascade activation, indicating the role of these fibroblasts at the interface with the immune system. CF1 showed enrichment for ECM-related pathways, including ECM organization and elastic fiber-associated pathways, supporting implication in ECM structural programs. CF2 was enriched for smooth muscle contraction together with ECM organization and ECM proteoglycans, suggesting important roles in ECM remodeling. CF3 markers were enriched for WNT signaling alongside ECM-related pathways (including ECM organization and ECM proteoglycans), supporting a WNT-modulating fibroblast subset with matrix-associated features. As expected, the transcriptional program associated with proliferating fibroblasts in CF4 exhibited strong enrichment for cell-cycle regulators (cell cycle, M phase, and cell-cycle checkpoints).

### 2.2. Matrisome Category Profiling Reveals Subcluster-Specific ECM Programs

Cardiac fibroblasts are the major source of ECM in the heart. Because the relative abundance of fibroblast subclusters differs between neonatal and adult hearts (Figure 1D), we then asked whether these subclusters also differ in ECM-related transcriptional programs. Matrisome categories were defined using the Naba/Matrisome Project gene sets, a list of ECM and ECM-associated genes [11]. These include the core matrisome (collagens, ECM glycoproteins, proteoglycans) and matrisome-associated genes (ECM regulators, ECM-affiliated proteins, secreted factors).

**Figure 3:**
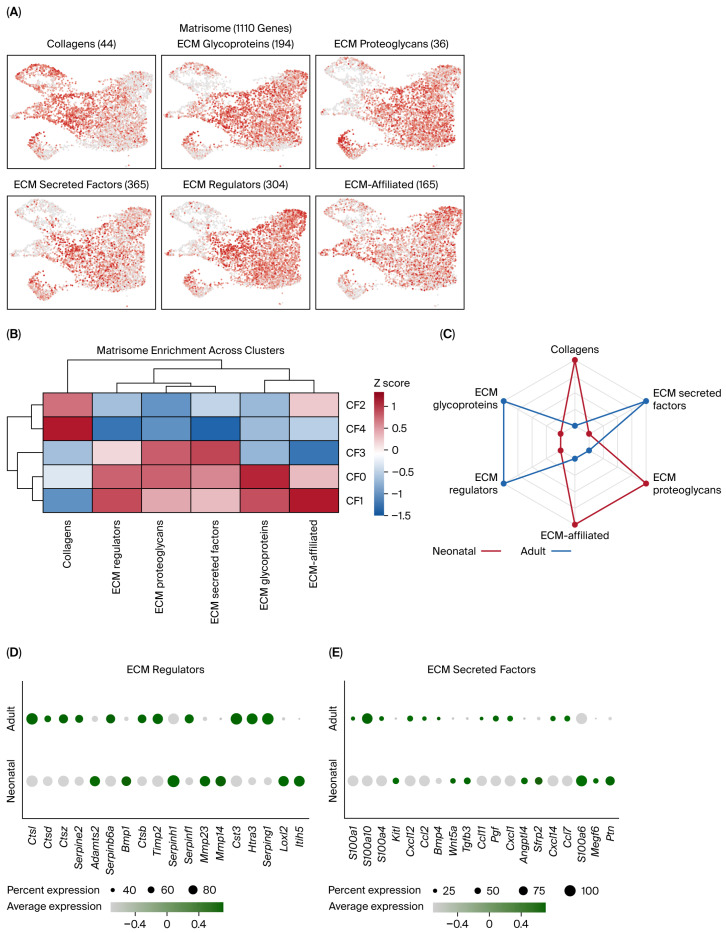
Matrisome category profiling reveals subcluster-specific ECM programs. (**A**) UMAP feature plots of matrisome categories across cardiac fibroblasts. Matrisome categories were defined using Naba/Matrisome Project gene sets (collagens, ECM glycoproteins, proteoglycans, ECM secreted factors, ECM regulators, and ECM-affiliated genes) and visualized using the Seurat AddModuleScore function; category sizes are indicated in the figure. (**B**) Heatmap showing mean matrisome category enrichment across CF subclusters, with hierarchical clustering of CF subclusters and matrisome categories. (**C**) Radar plots show mean module scores, calculated at the single-cell level and then summarized by age group within each fibroblast subcluster. (**D**,**E**) Dot plots showing expression of the top differentially expressed genes between neonatal and adult cardiac fibroblasts within the matrisome-associated categories ECM regulators (**D**) and ECM secreted factors (**E**). Dot size indicates the percentage of cells expressing each gene, and color intensity represents average expression.

Matrisome category scores were calculated for each fibroblast subcluster using the AddModuleScore function in Seurat and visualized on UMAP (Figure 3A). These scores showed clear spatial patterning across the CF landscape, indicating that ECM-related programs vary across fibroblast subclusters. CF2 and CF4 matrisome categories were closely associated and were characterized by strong enrichment for collagen programs (Figure 3B). In contrast, CF0 and CF1 showed the opposite pattern, with lower collagen enrichment and higher scores across categories, implicating ECM regulators and ECM glycoproteins. CF1 also showed strong enrichment for ECM-affiliated genes. CF3 displayed a distinct profile, with enrichment for ECM proteoglycans and ECM secreted factors.

Importantly, matrisome category profiles differed strongly between neonatal and adult fibroblasts (Figure 3C). Neonatal fibroblasts showed higher scores for core matrisome categories, particularly collagens and ECM proteoglycans, whereas adult fibroblasts showed higher scores for ECM glycoproteins, ECM regulators, and ECM secreted factors.

**Figure 4:**
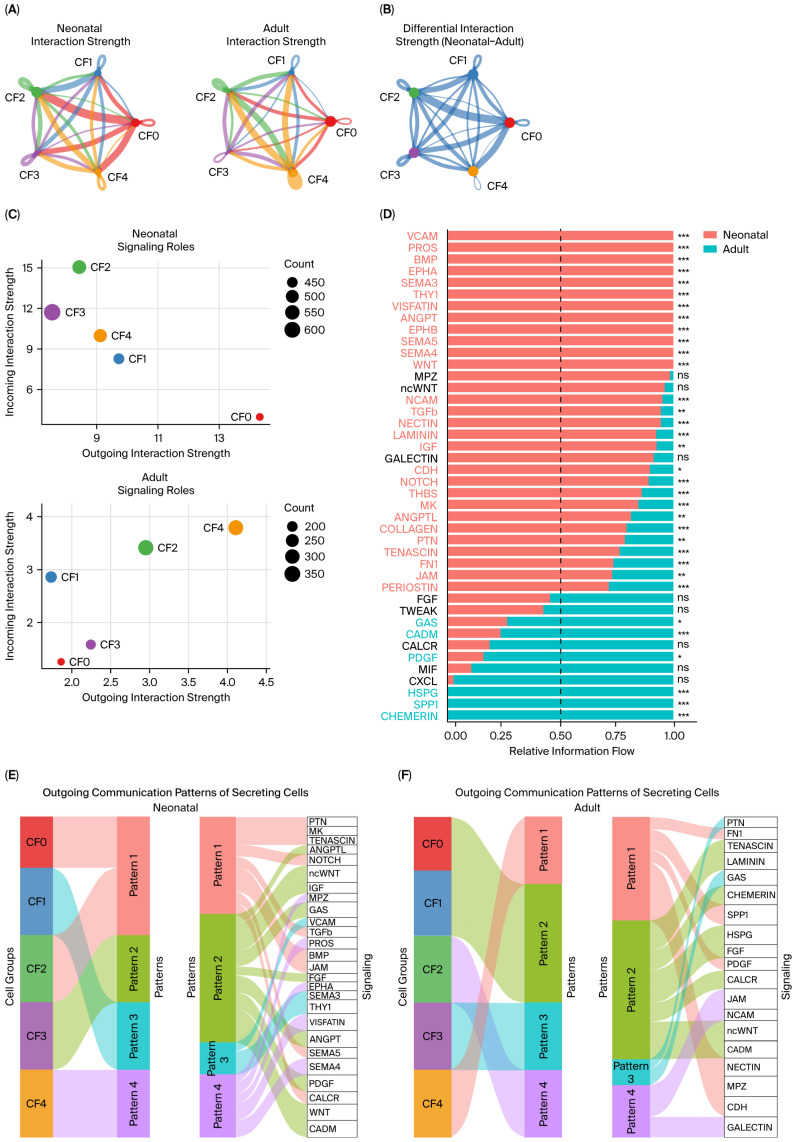
Fibroblast subcluster communication networks differ between neonatal and adult hearts. CellChat was used to identify ligand–receptor interactions and construct predicted communication networks among CF subclusters in neonatal and adult hearts. (**A**) Interaction strength networks among CF subclusters. Networks were computed separately for neonatal and adult hearts. (**B**) Differential interaction strength network (neonatal minus adult). Blue edges indicate stronger interactions in neonatal hearts; red edges indicate stronger interactions in adult hearts. (**C**) Signaling-role analysis showing outgoing (sender) and incoming (receiver) interaction strength for each CF subcluster in neonatal and adult hearts. (**D**) Ranked signaling pathways contributing to total information flow within the CF subcluster network, shown for neonatal and adult hearts; significance is indicated in the plot. (**E**,**F**) River plots showing the contribution of CF subclusters to each outgoing pattern and the signaling pathways associated with each pattern in neonatal (**E**) and adult (**F**) hearts. * *p* < 0.05, ** *p* < 0.01, *** *p* < 0.001, ns = not significant.

Gene-level analysis showed that adult fibroblasts are relatively enriched for ECM regulators linked to proteolysis and matrix turnover (e.g., cathepsins *Ctsl*/*Ctsd*/*Ctsz*, *Serpine2*, *Timp2*, Htra3) together with secreted chemokines (e.g., *Ccl2*, *Ccl7*, *Cxcl1*, *Cxcl14*), consistent with an immune-interfacing stromal environment. In contrast, neonatal fibroblasts preferentially express ECM regulators involved in matrix organization and remodeling (e.g., *Adamts2*, *Serpinh1*, *Mmp14*, *Loxl2*, *Itih5*) along with secreted developmental/tissue-repair factors (e.g., *Wnt5a*, *Sfrp2*, *Kitl*, *Ptn*, *Tgfb3*, *Angptl4*). These findings suggest that age-dependent ECM specialization is coupled to distinct paracrine signaling environments; therefore, we then examined fibroblast communication networks (Figure 4).

### 2.3. Fibroblast Subcluster Communication Networks Differ Between Neonatal and Adult Hearts

We then investigated communication across CF subclusters, identifying ligand–receptor interactions and mapping predicted signaling networks [12]. Ultimately, we compared networks between neonatal and adult hearts. Interaction strengths, computed separately for neonatal and adult hearts, showed extensive predicted communication between CF subclusters (Figure 4A). The differential network analysis depicted in Figure 4B demonstrates that predicted interaction strength was globally higher in neonatal hearts as compared to what was observed in the adult heart (Figure 4B; edges are defined as neonatal compared to adult; blue edges indicate stronger interactions in neonatal hearts). Consistent with this, the total inferred interaction strength was 48.63 in neonatal fibroblast networks and 12.80 in adult fibroblast networks. Altogether, these results indicate a widespread reduction in predicted fibroblast subcluster communication strength in the postnatal heart undergoing maturation (Figure 4B).

Signal analysis indicated that CF subclusters adopt distinct roles as sender or receiver in neonatal vs. adult hearts (Figure 4C). In neonatal hearts, CF0 showed the highest outgoing signaling connections with relatively low incoming signals, consistent with its role as a dominant signaling hub. In contrast, CF2 and CF3 received mainly incoming signals. In the adult heart, overall signaling levels were reduced, and proliferating fibroblasts in CF4 showed important outgoing signaling. CF4 also received substantial incoming signaling, indicating a shift in network centrality toward CF4 in the adult heart. Interestingly, CF0, while being the largest subcluster in the adult heart, displayed low outgoing signaling, highlighting that subcluster size and communication are not necessarily aligned. Finally, CF2 remained a prominent signal integrator in both neonatal and adult networks.

We then examined which signaling pathways were predicted to contribute most strongly to communication between fibroblast subclusters and to the overall interaction network (information flow) (Figure 4D). In neonatal hearts, ECM and adhesion-related pathways accounted for a large fraction of fibroblast communication, including LAMININ, COLLAGEN, FIBRONECTIN 1, TENASCIN, THROMBOSPONDIN, and PERIOSTIN. Development-related pathways, such as NOTCH, EPHRINS, BMP, and ANGPTL, were also overrepresented (Figure 4D). In contrast, a smaller set of pathways showed higher information flow in adult hearts, including SPP1, HSPG, CHEMERIN, GAS, CADM, and PDGF, consistent with a shift from ECM/adhesion dominant signaling toward more chemotactic signaling in the adult fibroblast network (Figure 4D).

We then used CellChat pattern analysis to decompose outgoing communication into a small number of dominant signaling modules and define which fibroblast subclusters contribute to each module (Figure 4E,F). In neonatal hearts, outgoing signaling is organized into four patterns with distinct subcluster contributions (Figure 4E). Pattern 1 was driven by CF0 and CF2 and included PTN, MK, TENASCIN, NOTCH, TGFB, BMP, JAM, SEMA5, and CALCR. Pattern 2 was dominated by CF3 and included ANGPTL, ncWNT, IGF, GAS, FGF, ANGPT, PDGF, WNT, and CADM. Pattern 3 was driven by CF1 and included VCAM, SEMA3, and THY1. Finally, Pattern 4 was driven by CF4 and included SEMA4, VISFATIN, EPHA, PROS, and MPZ. In adult hearts, outgoing signaling is also organized into four patterns, but with a different distribution of subcluster contributions and pathway modules (Figure 4F). Pattern 1 was driven by CF4 and included FN1, SPP1, PDGF, NECTIN, MPZ, and CDH. Pattern 2 was driven by CF0 and CF1 and included TENASCIN, LAMININ, CHEMERIN, HSPG, FGF, CALCR, ncWNT, and CADM. Pattern 3 was dominated by CF3 and included PTN and GAS, whereas Pattern 4 was driven by CF2 and included JAM, NCAM, and GALECTIN (Figure 4F). Together, these findings indicate that postnatal maturation alters not only the overall predicted interaction strength among fibroblast subclusters (Figure 4B–D) but also the dominant source subclusters and the pathway composition of fibroblast outgoing signaling (Figure 4E,F). Overall, neonatal fibroblast outgoing signaling was enriched for growth factor and developmental signaling, whereas adult signaling shifted toward ECM/adhesion and remodeling modules.

### 2.4. Adult MI Partially Recapitulates Neonatal Fibroblast Programs, Whereas Neonatal MI Exhibits an Additional Neonatal-Specific Injury Program

**Figure 5:**
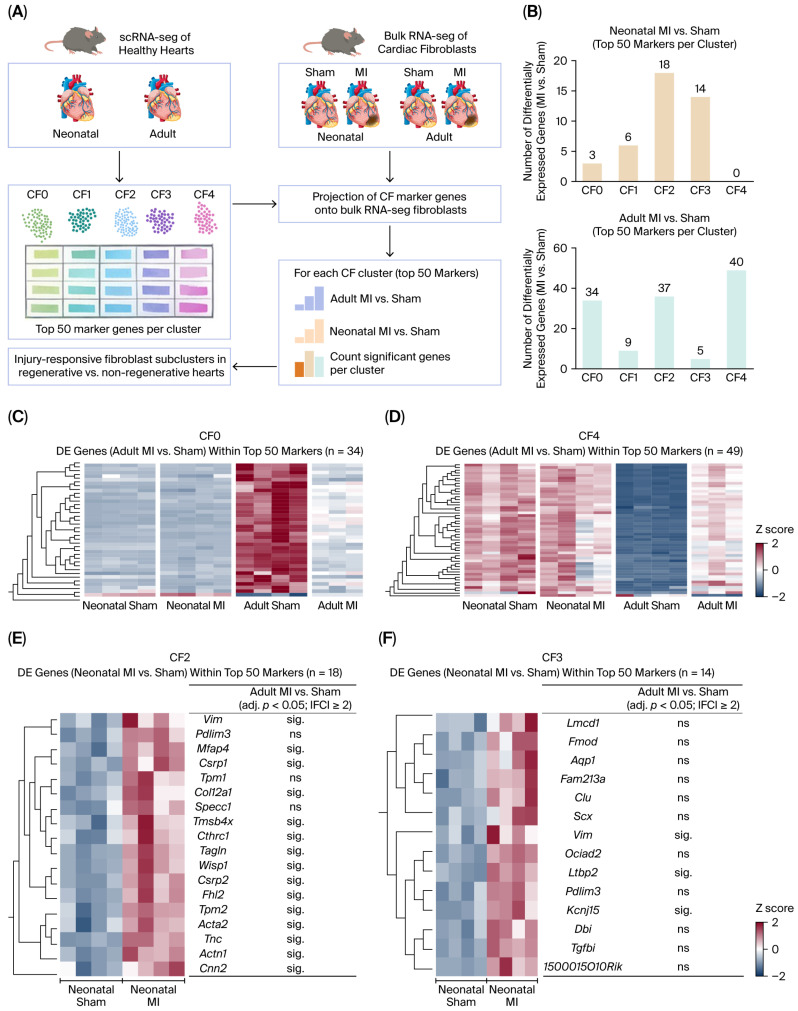
Neonatal and adult hearts engage distinct fibroblast subcluster programs after MI. (**A**) Schematic overview of the analysis workflow. Top 50 marker genes for each cardiac fibroblast subcluster (CF0–CF4; Supplementary Table S1) identified in our healthy scRNA-seq reference were projected onto an independent bulk RNA-seq dataset of cardiac fibroblasts collected 3 days after myocardial infarction (MI) or sham surgery in neonatal and adult mice. Differential expression (DE) of subcluster marker genes was assessed for MI vs. sham in each age group. (**B**) Bar plots showing the number of DE genes (MI vs. sham) among the top 50 marker genes for each CF subcluster in neonatal (**top**) and adult (**bottom**) fibroblasts. DE was defined as adj. *p*-value < 0.05 and |FC| ≥ 2. (**C**,**D**) Heatmaps illustrating adult MI responsive changes within CF marker programs. Genes shown represent the subset of each subcluster’s top 50 markers that were DE in adult MI vs. sham: CF0 (**C**; n = 34) and CF4 (**D**; n = 49). Expression is displayed across neonatal and adult sham/MI conditions (group labels indicated). (**E**,**F**) Recapitulation of neonatal injury response genes in adults. For CF2 (**E**) and CF3 (**F**), heatmaps show genes from each subcluster’s top 50 marker set that were DE in neonatal MI vs. sham (CF2: n = 18; CF3: n = 14). The table on the right indicates whether each neonatal MI responsive gene is also significant in adult MI vs. sham under the same criteria (adj. *p* < 0.05; |FC| ≥ 2), classified as sig or ns.

To assess how fibroblast subcluster programs were affected by injury at the onset of the disease, we projected the top 50 marker genes of each CF subcluster (CF0–CF4; Supplementary Table S1) onto an independent bulk RNA-seq dataset characterizing the cardiac fibroblast response 3 days after MI in either neonatal or adult mice (Figure 5A) [16]. Because this analysis was performed on an independent bulk RNA-seq dataset generated from sorted cardiac fibroblasts (CD90^+^ fibroblasts), it provides a gene program-level comparison rather than single-cell state resolution and therefore cannot distinguish program activation within fibroblasts from changes in fibroblast-state abundance. For each subcluster, we quantified marker expression and identified differentially expressed (DE) genes in MI vs. sham at each developmental age.

Clusters were differently involved during the response to injury in the regenerative (neonatal) and non-regenerative (adult) hearts (Figure 5B). In neonatal fibroblasts, MI induced transcriptional changes predominantly in marker genes associated with CF2 and CF3 (18 and 14 DE genes, respectively), indicating that these programs contribute strongly to the neonatal injury response. CF0 and CF1, on the contrary, showed minimal changes, and CF4 marker expression was not significantly altered. In sharp contrast, adult fibroblasts displayed a broader injury response, with substantial differential expression among markers from CF0, CF2, and CF4 (34, 37, and 49 DE genes, respectively), while CF3 showed minimal changes (Figure 5B). Thus, CF2 markers were induced in both neonatal and adult hearts, whereas the key age-dependent differences were driven by CF3 (activated mainly in neonatal) and CF0/CF4 (activated mainly in adult); CF1 showed little change in either group.

We then assessed whether the adult MI response recapitulates features of neonatal-associated fibroblast programs. We focused on marker genes that were differentially expressed in adult MI versus sham (DEGs in CF0, CF2, and CF4). Heatmaps depicting DEG subsets in CF0 (*n* = 34) and CF4 (*n* = 49), displayed across neonatal and adult conditions, showed that MI reversed the adult transcriptional programs toward a neonatal expression pattern (Figure 5C,D). Notably, the adult CF4 program defined by cell-cycle gene activation is consistent with the robust induction of proliferation after infarction. A similar, but less pronounced, neonatal-like shift was also observed for CF2 marker genes (Supplementary Figure S3).

Finally, we determined whether injury-response genes in neonates are recapitulated in adults. In CF2 (contractile/ECM-remodeling), most genes were similarly modulated following MI, supporting a shared injury-responsive component within the CF2 program across ages (Figure 5E). Consistently, CF2 was characterized by a contractile/ECM-remodeling signature (*Postn*, *Acta2*, *Tagln*, and *Tnc*; Figure 2A).

Interestingly, the majority of neonatal MI-responsive genes in CF3 were not significantly modulated in adulthood (Figure 5F). As mentioned above, CF3 includes a series of genes implicated in WNT signaling. Specifically, *Wif1*/*Fmod*-associated WNT-modulating program with potential anti-fibrotic properties could represent a primary target for inducing regenerative repair in the damaged adult heart.

## 3. Discussion

In this study, we defined five cardiac fibroblast subclusters with distinct age-dependent representation and differences in ECM and intercellular communication programs. Overall, neonatal fibroblasts show stronger core matrisome signatures and more extensive fibroblast-fibroblast communication, whereas adult fibroblasts are dominated by a more homeostatic ECM state with reduced information flow and a shift toward a narrower set of pathways, including immune/chemotactic signaling. Finally, comparison with an independent MI bulk RNA-seq dataset suggests that adult injury only partially reactivates neonatal-associated programs and lacks a neonatal-specific injury module, supporting fundamentally divergent stromal responses underlying regenerative versus fibrotic repair.

Loss of regenerative capacity after birth is a defining feature of mammalian cardiac biology. Classic studies demonstrated that neonatal mouse hearts can regenerate after injury within a narrow postnatal window, whereas this capacity is rapidly lost during maturation [1]. Our data extend this concept by providing a fibroblast-centered view of postnatal maturation. In particular, the stromal compartment is not a uniform “fibroblast population”, but instead comprises discrete transcriptional and functional states whose abundance differ markedly between neonatal and adult hearts. This observation is consistent with the published single-cell RNASeq data showing that fibroblasts diversify and transition through dynamic states during cardiac injury and disease [17]. Using matrisome [11] category scoring, we observed an age-dependent divergence in fibroblast ECM program architecture. Neonatal fibroblasts showed higher scores for core matrisome components, particularly collagens and proteoglycans, whereas adult fibroblasts exhibited comparatively greater enrichment of matrisome-associated categories, including the ECM regulators and ECM secreted factors. Gene-level analysis indicates that these differences are driven by distinct molecular programs. Adult fibroblasts preferentially expressed ECM regulators associated with proteolysis and matrix turnover (e.g., cathepsins *Ctsl*/*Ctsd*/*Ctsz*, *Serpine2*, *Timp2*, *Htra3*) together with chemokine-like secreted factors (*Ccl2*, *Ccl7*, *Cxcl1*, *Cxcl14*), consistent with an immune-interfacing stromal environment [18,19]. In contrast, neonatal fibroblasts were enriched for ECM regulators involved in matrix organization and remodeling (*Adamts2*, *Serpinh1*, *Mmp14*, *Loxl2*, *Itih5*) [16,20] along with developmental and tissue-repair-associated secreted factors, including *Wnt5a*, *Sfrp2*, *Kitl*, *Ptn*, and *Tgfb3* [19]. Biologically, this age-dependent divergence may reflect distinct ECM requirements in developing versus mature myocardium. Importantly, increased expression of core matrisome genes in neonatal fibroblasts should not be interpreted exclusively as a regeneration-specific signature. Because neonatal hearts are undergoing active growth and tissue assembly, this profile likely reflects, at least in part, developmental ECM formation. At the same time, developmental matrix properties may create a stromal environment that is more permissive for adaptive repair. Therefore, the present data support an association between neonatal ECM programs and the regenerative context, but do not by themselves distinguish developmental matrix production, injury-related remodeling, and truly regeneration-supportive matrix features. Neonatal hearts undergo rapid growth and remodeling and may therefore require robust structural ECM production and turnover, whereas adult hearts may rely more heavily on regulatory mechanisms that tune matrix organization, stiffness, and growth factor availability. While our data do not directly measure ECM deposition or tissue mechanics, they provide a transcriptomic rationale for distinct matrix remodeling processes that may occur in regenerative and non-regenerative contexts. Notably, subcluster annotation integrating marker genes and pathway enrichment further supports this functional stratification, with CF1 showing an ECM structural/elastic fiber-associated program, CF2 a contractile/ECM-remodeling program, CF3 a Wnt-modulating, matrix-regulatory signature, and CF4 a proliferative (cell-cycle) program.

Beyond ECM production, fibroblast function is shaped by paracrine and juxtacrine communication [21,22,23]. Using CellChat, we observed a global reduction in predicted interaction strength and information flow in adult fibroblast networks compared with neonatal networks, accompanied by changes in pathway composition and sender/receiver roles. CellChat infers intercellular communication probabilities by integrating single-cell expression with curated ligand–receptor knowledge and network analysis, enabling systematic comparison of signaling architecture across conditions [12]. Although ligand-receptor inference is inherently predictive and depends on database coverage and modeling assumptions, comparative analyses can be informative, especially when interpreted at the level of modules and pathway families rather than single interactions. In our dataset, neonatal fibroblast networks were dominated by ECM/adhesion-related programs, consistent with an actively remodeling developmental matrix environment. In contrast, adult networks showed reduced overall information flow with relatively higher contribution of a more restricted set of adult-enriched signaling pathways (Figure 4). These findings should be interpreted as inferred signaling architecture and candidate pathway modules rather than direct functional evidence. Given that inflammatory pathways such as NFKB drive endothelial-to-mesenchymal transition and promote profibrotic stromal expansion in cardiovascular disease [24,25], the observation that adult cardiac fibroblasts adopt an immune/chemotactic communication profile [26,27] suggests that altered fibroblast–endothelial–immune crosstalk is likely to be an important determinant of leukocyte recruitment and chronic fibrotic remodeling in the non-regenerative heart.

A key translational question is whether adult infarct repair fails because regenerative programs cannot be engaged in CFs, or because they are engaged but insufficient to drive effective repair. By projecting fibroblast subcluster marker programs onto an independent bulk RNA-seq MI dataset, we found evidence for partial overlap. MI in the adult heart engages a contractile/ECM-remodeling program overlapping with that seen in the neonatal heart. However, MI in neonates activates an additional neonatal-specific injury program that is weak or absent in adults (CF3). In addition, MI in adults is associated with strong activation of the cycling/proliferative program (CF4), consistent with robust induction of cell-cycle-associated markers early after infarction. This conceptual model aligns with prior single-cell studies showing that fibroblasts pass through sequential states after MI (including proliferative and matrix-remodeling phases), but that the timing, composition, and persistence of these states can differ across contexts and may shape whether repair remains adaptive or becomes maladaptive [17]. Importantly, our projection analysis does not prove lineage equivalence between neonatal and adult subclusters; rather, it provides a gene program-level connection that motivates future mechanistic experiments to test whether inducing elements of the neonatal-specific module in adult fibroblasts can alter healing trajectories.

An interesting hypothesis emerging from our data is that the neonatal-enriched CF3 state may contribute to a pro-regenerative stromal niche by locally tuning Wnt signaling. Because CF3 is characterized by expression of Wif1, Rspo3, and Fmod, one possible interpretation is that these fibroblasts help maintain a signaling environment that preserves cardiomyocyte plasticity and delays full terminal maturation, thereby indirectly supporting cardiomyocyte cell-cycle competence after injury. This is consistent with evidence that cardiomyocyte proliferation and maturation are tightly coupled processes and that immature or partially dedifferentiated states, supported by specific stromal and ECM cues, favor regenerative responses [5,9,28,29]. This idea remains speculative, as our study does not directly test fibroblast-to-cardiomyocyte signaling, but it aligns with prior work showing that postnatal fibroblast state switching influences cardiomyocyte maturation [5,30] and with the broader literature indicating that cardiomyocyte maturation and regenerative competence are tightly linked processes [9,31]. In this context, the weak induction or absence of the CF3 program in adult injury may reflect not only loss of a fibroblast subset, but loss of a developmental, Wnt-modulating matrix program permissive for regenerative repair. Given the CF3 signature, an important next step will be to test whether failure to engage this Wnt-modulating, matrix-regulatory program contributes causally to the adult injury response, for example, by assessing how experimental enhancement of CF3-like Wnt modulation in adult stroma impacts cardiomyocyte proliferative capacity and maturation status in vivo.

One limitation of the present scRNA-seq design is the pooling of cells prior to 10x Genomics library preparation. Although four hearts per age group were processed individually through dissociation and FACS, inter-animal variability could not be formally assessed at the individual sample level. A further limitation of the present study is that the CF3 program was defined at the transcriptomic level only. Therefore, the spatial localization, protein-level expression, and functional relevance of CF3-associated markers such as Wif1, Rspo3, and Fmod remain to be established by orthogonal validation approaches.

In summary, we provided a fibroblast-focused single-cell framework that distinguishes regenerative neonatal from non-regenerative adult hearts by fibroblast state composition, matrisome specialization, and communication network architecture. This framework serves as a resource to interpret age-dependent stromal responses and nominates fibroblast programs and signaling modules that may be leveraged to promote adaptive repair while limiting maladaptive fibrosis after MI. Determining which neonatal stromal signals are functionally required for regenerative remodeling will be essential for designing interventions that modulate fibroblast behavior without compromising scar integrity and risking rupture.

## 4. Materials and Methods

### 4.1. Animal Experiments

Mouse studies were authorized by the Government Veterinary Office (Lausanne, Switzerland) and conducted under the institutional guidelines of the University of Lausanne, in compliance with Swiss animal welfare legislation.

### 4.2. Nonmyocyte Cell Isolation from Adult Mouse Hearts

Adult mice received 100 U Liquemin 15 min before sacrifice to prevent blood clotting. Hearts were excised while beating, immediately transferred to ice-cold PBS, and aortas were cannulated for retrograde perfusion. The hearts were first perfused with Ca^2+^/Mg^2+^-free HBSS to remove residual blood, followed by perfusion with an enzymatic digestion solution (Pierce Primary Cardiomyocyte Isolation Kit; Thermo Fisher Scientific, Waltham, MA, USA; cat. 88281). After each enzyme perfusion, hearts were incubated for 10 min at 37 °C. This perfusion–incubation cycle was repeated 3–4 times. Atria and large vessels were removed, and ventricles were minced and gently triturated in DMEM (15–20 pipetting steps) to obtain a single-cell suspension. The suspension was filtered through a 300 µm mesh, and cardiomyocytes were depleted via low-speed centrifugation (30 g, 5 min). The supernatant containing nonmyocytes was collected, passed through a 40 µm strainer, and the nonmyocyte cells were pelleted via centrifugation (800 rpm, 5 min). Final cell pellets were resuspended in DMEM supplemented with 10% fetal bovine serum.

### 4.3. Nonmyocyte Cell Isolation from Neonatal Mouse Hearts

Neonatal C57BL/6 mice were euthanized at postnatal day 3, and hearts were collected while beating. Atria and large vessels were removed, and ventricles were minced into ~1–3 mm^2^ fragments and washed twice in ice-cold HBSS. Minced tissue was pooled (4 hearts) and incubated in enzymatic digestion solution (Pierce Primary Cardiomyocyte Isolation Kit; Thermo Fisher Scientific; cat. 88281) at 37 °C with shaking for 30 min. The enzyme solution was then removed, the tissue was washed in ice-cold HBSS, and the suspension was passed through a 40 µm cell strainer. Complete medium was added, and the sample was gently triturated (10–20 pipetting steps) to generate a single-cell suspension. During subsequent flow cytometry, cardiomyocytes were depleted based on cell size by excluding large events during gating prior to sorting.

### 4.4. Fluorescence-Activated Cell Sorting (FACS)

Nonmyocyte suspensions were enriched via FACS to exclude leukocytes and endothelial cells. Cells were first labeled with anti-CD45 (BioLegend, San Diego, CA, USA; cat. 103112) and anti-CD31 (BioLegend, cat. 102408) for 30 min at 4 °C. Cells were washed twice in PBS containing 1% FBS and then stained with Calcein AM (BD Biosciences, San Jose, CA, USA; cat. 564061) for 10 min at 37 °C. After two additional washes in PBS with 1% FBS, DAPI was added immediately before sorting to identify nonviable cells. Calcein AM-positive, DAPI-negative cells lacking CD45 and CD31 expression were collected and used for single-cell RNA sequencing.

### 4.5. Single-Cell RNA-Sequencing

For each age group, hearts from four animals were processed individually through tissue dissociation and FACS. Cell suspensions were pooled within each age group immediately before 10x Genomics library preparation, generating one pooled library for P3 and one pooled library for P84. Cells were counted using a hemocytometer, and viability was assessed via trypan blue exclusion (>80%). Single-cell libraries were generated using the 10x Genomics Chromium platform (Chromium Next GEM Chip) following the manufacturer’s protocol [32]. Briefly, cells were encapsulated with barcoded gel beads in oil to generate single-cell droplets, and barcoded cDNA was pooled for downstream library construction. Libraries were quantified and quality-controlled using a Fragment Analyzer (Agilent Technologies, Santa Clara, CA, USA). Sequencing was performed on an Illumina HiSeq 4000 (HiSeq 3000/4000 SBS reagents; Illumina, San Diego, CA, USA). Base calling and demultiplexing were performed using bcl2fastq2 (v2.20, Illumina, San Diego, CA, USA), and primary processing was carried out with the Cell Ranger Gene Expression pipeline (v3.0.2, 10× Genomics) [33].

### 4.6. scRNA-Seq Data Analysis

Raw sequencing data were demultiplexed and converted to FASTQ files, and reads were aligned to the mouse reference genome (mm10) using STAR (v2.6.0c) [34]. Gene barcode count matrices were generated using Cell Ranger (10× Genomics; v1.3). Downstream analyses were performed in R using Seurat (v5.3) with default settings unless otherwise specified [33,34,35]. After standard quality control and normalization, dimensionality reduction was performed using Uniform Manifold Approximation and Projection (UMAP) implemented in Seurat (RunUMAP). Calculation of mitochondrial gene content in the reclustered fibroblast dataset showed overall low mitochondrial percentages (median 1.84%; 95% of cells ≤ 3.45%), consistent with generally high-quality cells. Cluster marker genes were identified with FindAllMarkers using the Wilcoxon rank-sum test and a minimum fraction of expressing cells of 0.25 (min.pct = 0.25). *p*-values were adjusted for multiple testing using Bonferroni correction. Gene expression patterns were visualized using FeaturePlot, DoHeatMap, and DotPlot.

Predicted cell–cell communication networks were inferred using CellChat parameters [12]. Communication probabilities were estimated from single-cell expression data combined with the curated CellChat ligand–receptor interaction database. CellChat analysis was performed on the reclustered fibroblast subset using default parameters, including the default population.size = FALSE setting. Pathway-level information flow was compared between neonatal and adult fibroblast networks using rankNetwith measure = “weight” and do.stat = TRUE, for which CellChat applies a paired Wilcoxon test for comparison between two datasets with matched cellular composition.

Matrisome scoring was performed using gene sets from the Matrisome Project described by Naba and colleagues [11], which provides a standardized annotation of ECM and ECM-associated genes. Categories include the core matrisome (collagens, ECM glycoproteins, and proteoglycans) and matrisome-associated genes (ECM regulators, ECM-affiliated proteins, and secreted factors).

### 4.7. Bulk RNA-Seq Data Analysis

Publicly available bulk RNA-seq data from neonatal and adult cardiac fibroblasts collected after myocardial infarction (MI) and sham surgery were obtained from an external study [1]. Processed expression data were imported into Qlucore Omics Explorer (base module v3.10; Qlucore, Sweden) for downstream analysis and normalized using the trimmed mean of M values (TMM) method implemented in Qlucore. Differential expression between MI and sham was assessed separately within each age group using an adjusted *p* value threshold of <0.05 and an absolute fold-change of at least 2. To relate injury responses to the fibroblast states defined in our scRNA-seq reference, we projected subcluster marker programs by testing the top marker genes for each CF subcluster (CF0–CF4) for differential expression in the bulk dataset and quantifying overlap and directionality across neonatal and adult conditions. Gene expression patterns across groups were visualized using heatmaps generated from the normalized expression matrix.

## Data Availability

The original contributions presented in this study are included in the article/Supplementary Material. Further inquiries can be directed to the corresponding author.

## Supplementary Materials

The following supporting information can be downloaded at: https://www.mdpi.com/article/10.3390/ijms27062709/s1.
