# Supplementary material for: Decoding Fibroblast Diversity Associated with the Postnatal Loss of Cardiac Regenerative Capacity
Source: Int J Mol Sci. 2026 Mar 16;27(6):2709. doi: 10.3390/ijms27062709 (PMC13027323; doi:10.3390/ijms27062709)
Supplement: Supplementary file 1 [file ijms-27-02709-s001.zip › Updated Supplemental Figures.pdf]

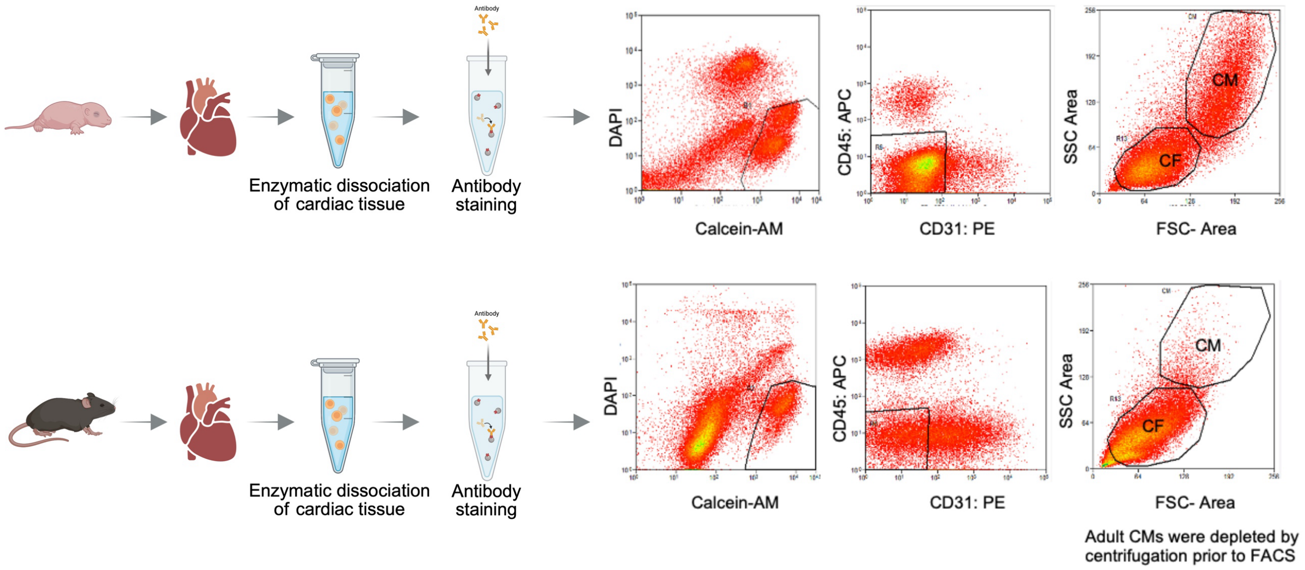


**Supplementary Figure S1. Cardiac nonmyocyte isolation and FACS gating strategy for neonatal and adult hearts.** Schematic overview of the workflow used to enrich viable cardiac fibroblasts for scRNA-seq from neonatal (P3) and adult (P84) mouse ventricles. Hearts were enzymatically dissociated to generate a single-cell suspension, followed by antibody staining against CD45 and CD31 and viability staining with Calcein-AM and DAPI. Representative flow cytometry plots show sequential gating: (i) selection of live, metabolically active cells (Calcein-AM$^{+}$/DAPI$^{-}$), (ii) exclusion of leukocytes and endothelial cells (CD45$^{-}$/CD31$^{-}$), and (iii) FSC/SSC-based separation of cardiomyocytes (CM) from cardiac fibroblasts (CF). In adult preparations, cardiomyocytes were depleted by centrifugation prior to FACS.

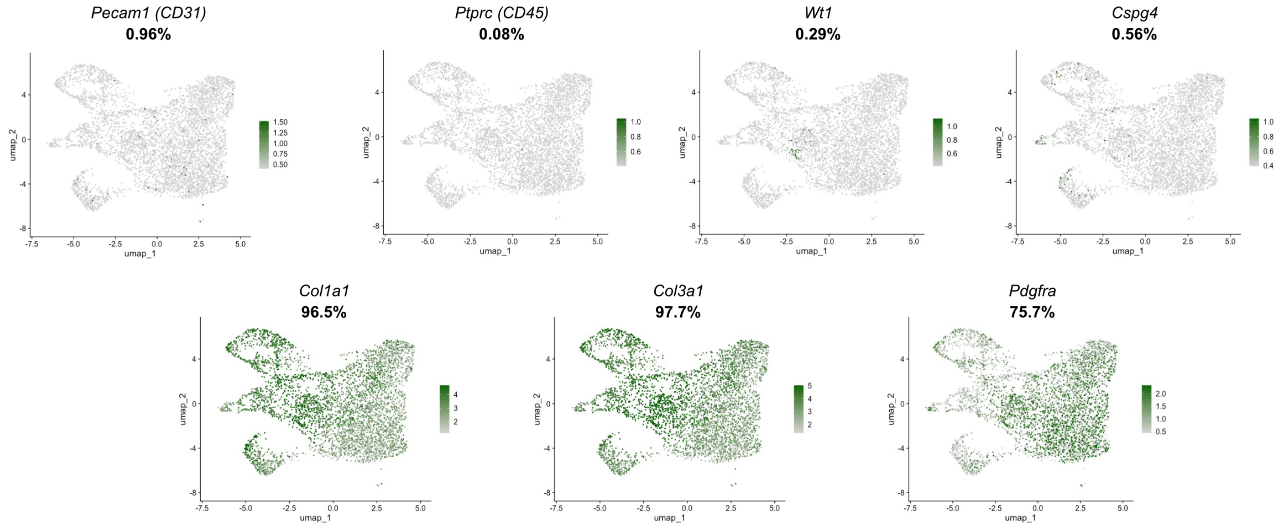


**Supplementary Figure S2. Validation of cell identity in the reclustered fibroblast dataset**. Feature plots showing expression of canonical endothelial (*Pecam1*/CD31), leukocyte (*Ptprc*/CD45), epicardial (*Wt1*), and pericyte (*Cspg4*) markers, together with canonical fibroblast markers (*Col1a1*, *Col3a1*, *Pdgfra*), in the pooled reclustered fibroblast dataset. Percentages indicate the proportion of marker positive cells in the reclustered fibroblast object.

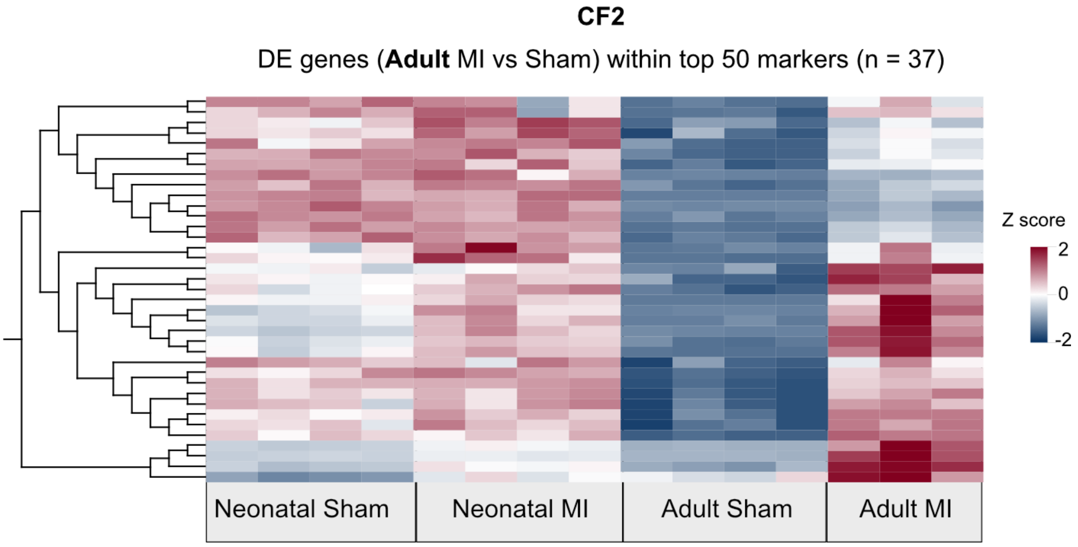


**Supplementary Figure S3. Adult MI responsive changes within the CF2 marker program.** Heatmap illustrating adult MI responsive changes within the CF2 marker program. Genes shown represent the subset of the top 50 CF2 marker genes (defined in the healthy scRNA-seq reference; Supplementary Table S1) that were differentially expressed in adult MI versus adult sham (n = 37). Expression is displayed across neonatal sham, neonatal MI, adult sham, and adult MI conditions (group labels indicated). Differential expression was defined as adj. P value < 0.05 and |FC| ≥ 2.
